# A carboxymethyl dextran-based polymeric conjugate as the antigen carrier for cancer immunotherapy

**DOI:** 10.1186/s40824-018-0131-0

**Published:** 2018-08-14

**Authors:** Jung Min Shin, Seok Ho Song, N. Vijayakameswara Rao, Eun Sook Lee, Hyewon Ko, Jae Hyung Park

**Affiliations:** 1 School of Chemical Engineering, Sungkyunkwan University, Suwon, 16419 Republic of Korea; 2 Department of Health Science and Technology, SAIHST, Sungkyunkwan University, Suwon, 16419 Republic of Korea; 3 School of Chemical Engineering, Sungkyunkwan University, Suwon, 440-746 Republic of Korea

**Keywords:** Carboxymethyl dextran, Cancer immunotherapy, Antigen delivery

## Abstract

**Background:**

Antigen-specific cytotoxic T lymphocytes (CTLs), which eliminate target cells bearing antigenic peptides presented by surface major histocompatibility complex (MHC) class I molecules, play a key role in cancer immunotherapy. However, the majority of tumors are not immunologically rejected since they express self-antigens which are not recognized by CTLs as foreign. To foreignize these tumors for CTL-mediated immunological rejection, it is essential to develop carriers that can effectively deliver foreign antigens to cancer cells.

**Methods:**

A polymeric conjugate, composed of a carboxymethyl dextran (CMD) as the backbone and ovalbumin (OVA) as a model foreign antigen, was prepared to investigate its potential as the antigen carrier for cancer immunotherapy.

**Results:**

An in vitro cellular uptake study showed that the conjugate was successfully taken up by TC-1 cervical cancer cells. When CMD-OVA was systemically administered to tumor-bearing mice, the strong fluorescence signal was observed at the tumor site over the whole period of time period, suggesting high tumor targetability of the conjugate. Compared to free OVA, CMD-OVA induced significantly higher antigen presentation at the tumor site.

**Conclusions:**

The CMD-OVA conjugate can effectively deliver the antigen to the tumor site, implying its high potential as the antigen carrier for cancer immunotherapy.

## Background

In recent years, cytotoxic T lymphocytes (CTL) have been extensively studied for their ability to destroy target cells bearing antigenic peptides presented by surface major histocompatibility complex (MHC) class I molecules [1–3]. Taking advantage of the unique functions of CTLs, chimeric antigen receptor (CAR)-T and adoptive cell therapy (ACT) have been used in clinical trials [4–6]. Although they have drawbacks such as high cost and limited use in autologous therapy, these therapeutic approaches are useful for cancer treatment.

Unfortunately, tumors can avoid CTL recognition because they have self-antigen on their surfaces. For CTL-mediated immunological rejection of tumors by foreignization, it is essential to develop carriers that can deliver foreign antigens to the cancer cells [7, 8]. To date, no significant effort has been devoted to the development of a tumor-specific intracellular delivery system for these antigens.

Polymeric conjugates with targeting ligands have been studied as drug carriers for cancer therapy. They accumulate passively at the tumor site via enhanced permeation and retention effect and undergo uptake via receptor-mediated endocytosis by tumor cells [9–14]. Among polymeric materials, considerable effort has focused on using carboxymethyl dextran (CMD) as a drug carrier for cancer therapy and imaging because of its high biocompatibility, excellent water solubility, and biodegradability [15–17]. In particular, owing to its multiple functional groups allowing for facile chemical modification, CMD has been extensively used to develop polymeric conjugates as the nanomedicines [18, 19].

**Fig. 1:**
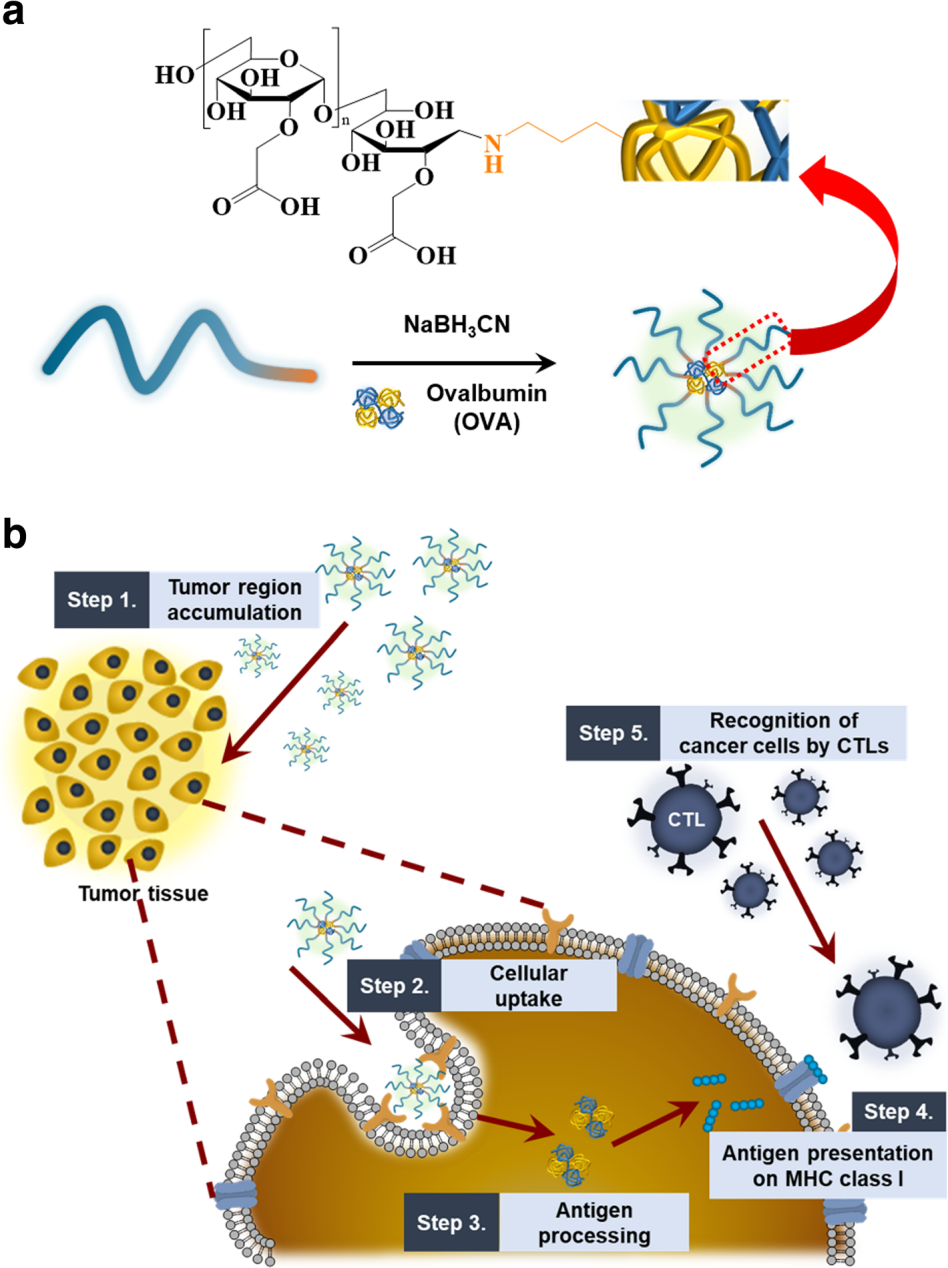
**a** Chemical structure of carboxymethyl dextran-ovalbumin (CMD-OVA) conjugate. **b** Schematic illustration of antigen presentation and immunological tumor rejection by cytotoxic T lymphocytes

Herein, in an attempt to foreignize the cancer cells, we prepared a CMD-based polymeric conjugate with ovalbumin (OVA) as a model foreign antigen (Fig. 1). Its uptake behavior by cancer cells have been assessed using confocal microscopy. After systemic administration of the conjugate into the tumor-bearing mice, it’s in vivo biodistribution was observed using the optical imaging technique. Also, in vivo antigen presentation was observed to estimate the potential of the conjugate as the antigen carrier for cancer immunotherapy.

## Methods

### Materials

CMD sodium salt (M_n_ = 10,000–20,000 Da), OVA, 1-ethyl-3-(3-dimethylaminopropyl)carbodiimide·hydrochloride (EDC·HCl), *N*-hydroxysuccinimide (NHS), sodium cyanoborohydride (NaBH_3_CN), and fluorescein isothiocyanate (FITC) were purchased from Sigma-Aldrich (St. Louis, MO, USA). The near-infrared fluorescent (NIRF) probe, cyanine 5.5 (Cy5.5), was purchased from Amersham Bioscience (Piscataway, NJ, USA). The water used in this study was prepared by an AquaMax-Ultra Water Purification System (Anyang, Republic of Korea). All other chemicals were prepared by commercial sources, and they were used as received without purification.

### Preparation of CMD-OVA conjugate

OVA was chemically affixed to CMD via reductive amination between the reducing end group of CMD and the amino group of OVA, in the presence of NaBH_3_CN as a reducing agent. Briefly, the CMD (82.65 mg) solution in 40 ml of borate buffer (pH 8.5, 0.4 M NaCl) was mixed with OVA (25 mg), dissolved in 1 mL of borate buffer (pH 8.5). Sodium cyanoborohydride (100 mg) was added to the reaction mixture, which was then allowed to stir for 5 days at 40 °C. The conjugate was obtained by dialysis using deionized water for 3 days in cellulose membrane bag (molecular weight cut off = 50 kDa), followed by lyophilization. Prior to use, the conjugate was stored at − 20 °C.

### Cellular uptake behavior of CMD-OVA conjugate

To investigate the internalization of the CMD-OVA conjugate in vitro, the conjugate was labeled with FITC. TC-1 cells (a cervical cancer cell line expressing the E7 protein of human papilloma virus type 16) were cultured in RPMI 1640 medium containing 10% (*v*/v) fetal bovine serum (FBS) and 1% (v/v) penicillin and streptomycin solution in a humidified cell culture incubator at 37 °C. The cells, seeded at 2 × 10^5^ cells/well in 6-well plates for 1 day, were treated either with FITC-labeled OVA or CMD-OVA in RPMI 1640 media without FBS for 3 h. The cells were then washed twice using PBS containing Ca^2+^ and Mg^2+^, and fixed using a 4% paraformaldehyde solution. The cellular uptake of the CMD-OVA conjugate was visualized using confocal laser scanning microscopy (CLSM) (TCS SP8 HyVolution, Leica Microsystems, Wetzlar, Germany) and quantified using flow cytometry (Guava easyCyte, EMD Millipore, Billerica, MA, USA).

### In vivo biodistribution

The conjugate was labeled with Cy5.5 as follows. In brief, the hydrazide derivative of Cy5.5 was reacted with carboxylic groups of CMD in the presence of EDC overnight in the dark. The reaction mixtures were then dialyzed (molecular weight cut off = 3 kDa) against deionized water to remove non-reacted Cy5.5 and EDC. The purified conjugate was stored at − 20 °C, prior to use. To observe the in vivo tumor-homing ability of the conjugate, a TC-1 flank tumor model was prepared by the subcutaneous inoculation of a cell suspension (1 × 10^6^ cells for each mouse) in RPMI medium (100 μL) into athymic nude mice (6-week-old, female). When the tumor volumes reached 150–200 mm^3^, 200 μL of physiological saline containing Cy5.5-labeled polymeric conjugate was injected into the tail vein of each mouse. The conjugate was visualized at predetermined time points by scanning the mice using a Lago X system (Spectral Instruments Imaging, Tucson, AZ, USA) with a 670 nm LED. The tumors and major organs (liver, heart, lung, spleen, and kidney) were excised from the mice at 48 h post-injection to quantitatively assess the ex vivo tissue distribution of the conjugate by measuring the average fluorescence intensity over the region of interest.

### In vivo antigen presentation

To prepare the tumor-bearing animal model, TC-1 cells (2 × 10^5^ cells for each mouse) were subcutaneously injected into C57BL/6 mice (8-week-old, female). After 10 days, each conjugate (OVA or CMD-OVA) was dissolved in saline containing 100 μg of OVA and was then systemically administered through the tail vein. At 24 h post-injection, the tumor tissue was removed, dissociated by a cell strainer, and washed twice with PBS. The biotin-conjugated anti-mouse pMHC-OVA_257–264_ antibody was used to stain D^b^/OVA_257–264_ complexes. For quantification of the in vivo OVA presentation, flow cytometry analysis was performed.

## Statistical analysis

The statistical significance of experimental results was assessed using one-way analysis of variance (ANOVA), and a *p* value < 0.05 was regarded as significant (indicated with an asterisk (*) in the corresponding figures).

## Results

### Preparation and characterization of the CMD-OVA conjugate

The CMD-OVA conjugate was prepared as a carrier that can effectively deliver antigens to cancer cells for CTL-mediated immunological rejection by foreignization. Of the various biocompatible polymers, CMD was chosen as the backbone due to its unique structure, high biocompatibility, excellent water solubility, and biodegradability. OVA was used as a model antigen, chemically attached to the reducing end group of the CMD chain via reductive amination in the presence of NaBH_3_CN. The OVA content of CMD-OVA was directly quantified by the bicinchoninic acid assay as 133 μg of OVA per 1 mg of CMD-OVA.

### In vitro cellular uptake

**Fig. 2:**
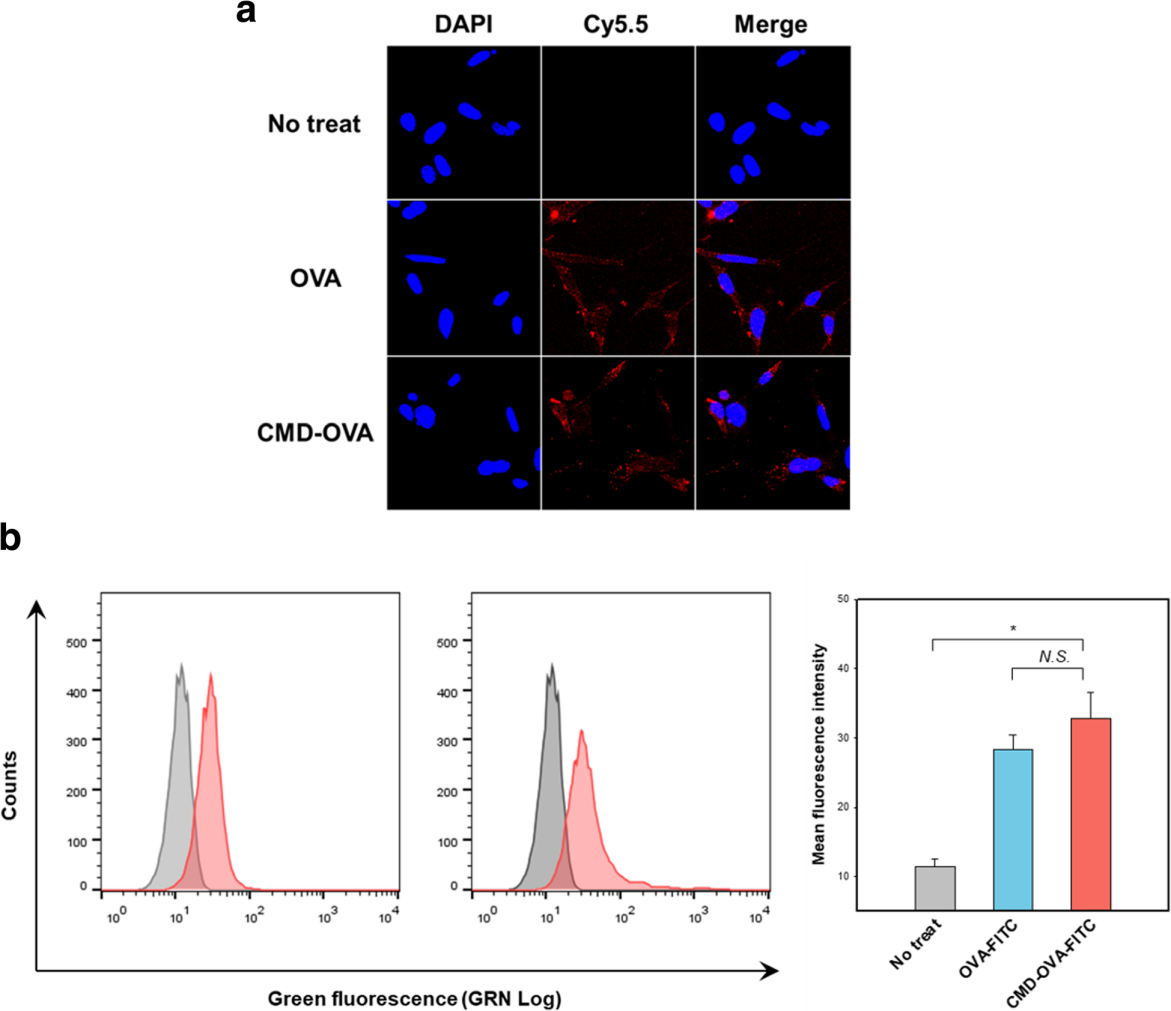
Cellular uptake behavior of CMD-OVA conjugate. **a** Representative confocal microscopic image. **b** Quantitative analysis using flow cytometry. Error bars represent the standard deviation (*n* = 3)

For CTL-mediated cell death, foreign antigens should be processed inside the target cells, followed by presentation on their surfaces as part of the MHC class I complex. Therefore, if the target cells do not internalize the conjugate, CTLs cannot identify and destroy them. To explore the cellular uptake of the conjugate in vitro, CMD-OVA was incubated with TC-1 cells, and its uptake was observed utilizing CLSM (Fig. 2a). CMD-OVA showed a similar intracellular fluorescence signal to the OVA-FITC control. The cellular uptake of the conjugate was also measured using flow cytometry (Fig. 2b). Quantitative analysis indicated that the amount of the conjugate, taken up by the cancer cells, were comparable to that of OVA.

### In vivo biodistribution of the CMD-OVA conjugate

**Fig. 3:**
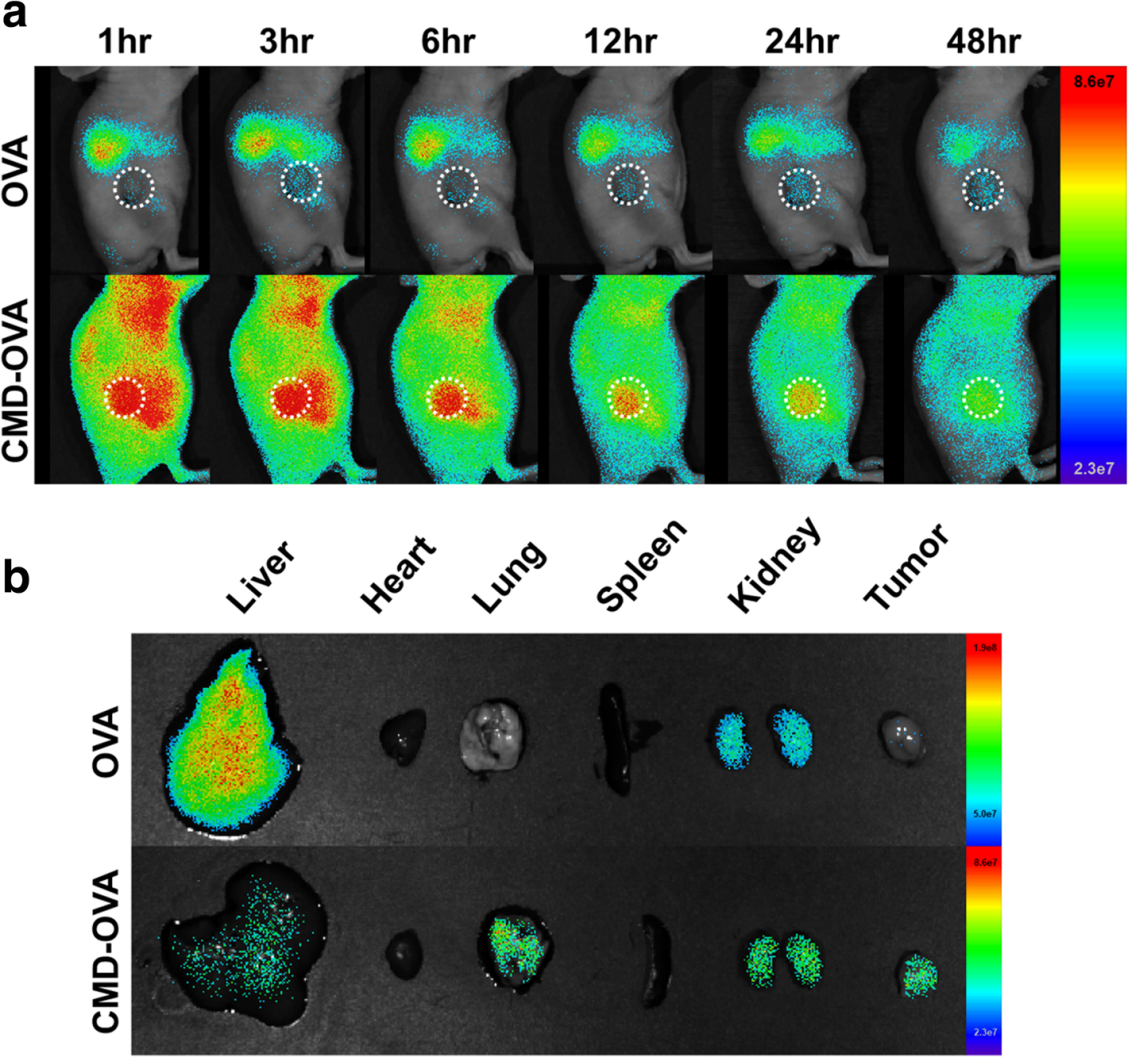
In vivo distribution of CMD-OVA conjugate. **a** Whole body near-infrared fluorescence (NIRF) image of CMD-OVA conjugate as a function of time. The white dotted line indicated tumor site. **b** Ex vivo NIRF images of tumor and major organs after 48 h

To examine the in vivo biodistribution, Cy5.5-labeled CMD-OVA or OVA was systemically injected into the TC-1 tumor-bearing mice. The fluorescence images of the tumor site were acquired by using a non-invasive optical imaging system. As shown in Fig. 3a, compared to OVA, the CMD-OVA conjugate exhibited stronger fluorescence signals in the entire bodies of the mice for the whole test period, implying prolonged circulation of CMD-OVA. It is worthy to note that strong fluorescence signals of CMD-OVA were observed at the tumor site (the white dotted line), whereas no significant signals were found for OVA. As demonstrated by ex vivo images of the major organs, most of OVA was accumulated in liver, where minimal amount of CMD-OVA was detected (Fig. 3b). This result implies that the CMD-OVA conjugate can effectively accumulate at tumor sites, thus possessing potential to effectively deliver the antigen to the tumor.

### In vivo antigen presentation of CMD-OVA conjugate

**Fig. 4:**
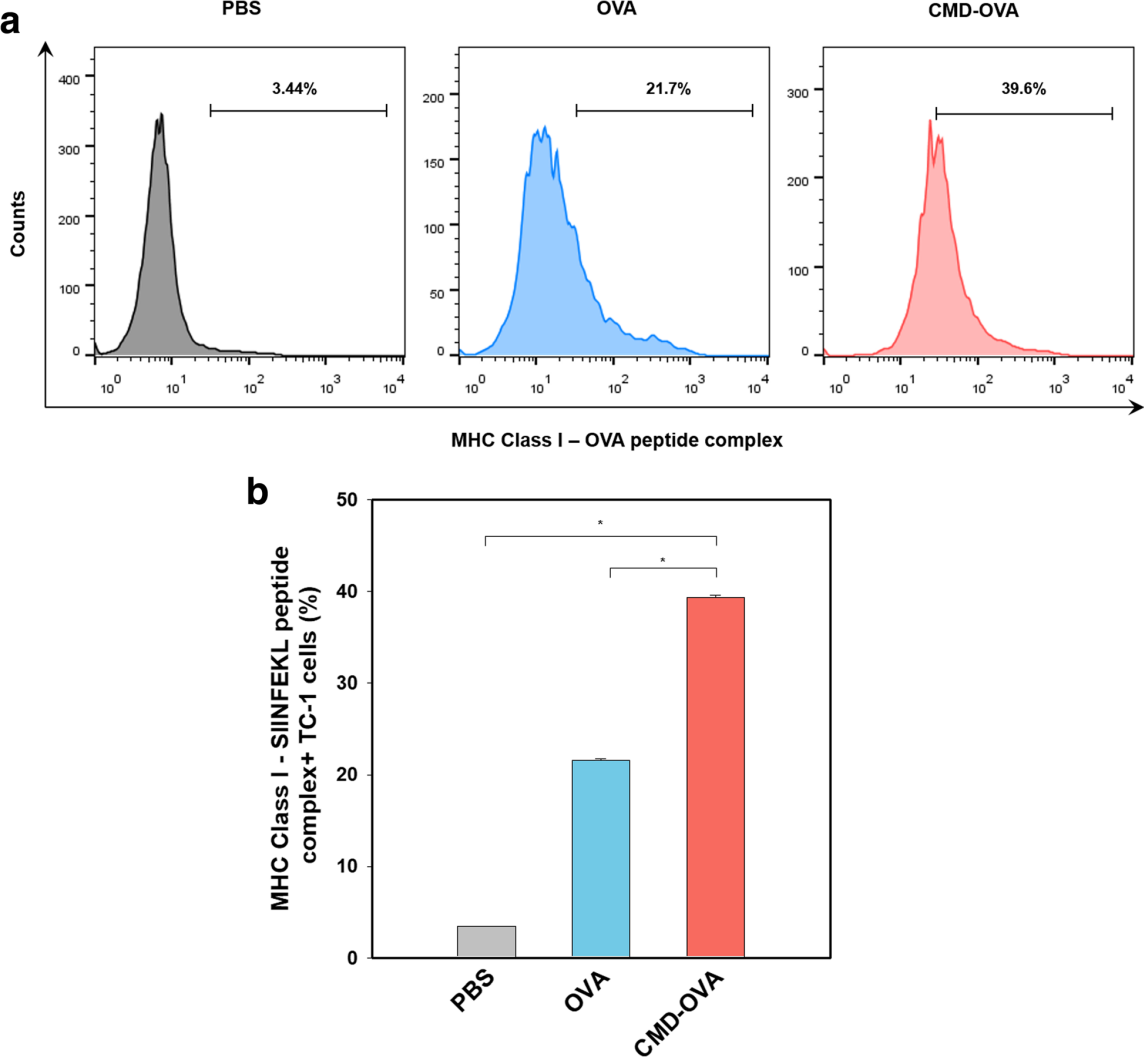
In vivo antigen presentation in a TC-1 tumor-bearing mouse model. **a** Quantification of the MHC class I-SIINFEKL^+^ in tumor cells. **b** Comparison of MHC class I-SIINFEKL^+^ cells in each sample group. Error bars represent the standard deviation (*n* = 3)

The OVA antigen presentation of TC-1 cells, treated with CMD-OVA or OVA, was measured by flow cytometry. After cell isolation from mice, TC-1 cells were stained with an anti-mouse MHC-OVA peptide (pMHC-OVA_257–264_) antibody, which specifically binds to mouse MHC class I-OVA peptide (OVA_257–264_) complexes. Relative OVA antigen presentation was quantified using flow cytometry (Fig. 4). The CMD-OVA conjugate induced much higher OVA_257–264_ presentation, compared with free OVA. Overall, these data suggest that the CMD-OVA conjugate is efficiently taken up by tumor cells, followed by the enhanced presentation of a passenger antigen (OVA)-MHC class I complex.

## Discussion

In recent years, owing to their unique characteristics, polymeric conjugates have gained attention for biomedical applications such as drug delivery and tissue engineering. Particularly, several conjugates have been approved by FDA because chemical attachment of biocompatible polymers to bioactive agents have been demonstrated to prolong their blood circulation, resulting in enhanced biological half-lives [20, 21].

In order to overcome current limitations of conventional chemotherapy, immunotherapeutic approaches have recently emerged based on immune checkpoint inhibitor, CAR T cell, and neoantigen [22–24]. For example, neoantigens derived from cancer cells have received attention because they are easily distinguished from self-antigens, leading to elimination of cancer cells by CTLs. In this study, the biocompatible CMD-based antigen carrier has been prepared to investigate its potential for cancer immunotherapy. From the in vitro cellular uptake study, it was found that the conjugate was effectively taken up by the cancer cells, implying that the conjugate can deliver the antigen into the intracellular compartments for antigen processing. After systemic administration into the tumor-bearing mice, the conjugate was efficiently accumulated at the tumor site, compared to free OVA. This high tumor targetability of the conjugate might account for significant antigen presentation in vivo (Fig. 4), which may facilitate recognition of the cancer cells by CTLs.

This foreign antigen delivery technology can be applied to various diseases. For example, for rheumatoid arthritis, activated macrophages are primarily responsible for inflammatory responses. If foreign antigens can be delivered to the activated macrophages, it may cause their apoptotic cell death by CTL-mediated immune responses, resulting in suppression of the symptoms by rheumatoid arthritis. Overall, this foreign antigen delivery technology based on the polymeric conjugates would be highly useful for treatments of various intractable diseases.

## Conclusion

The CMD-OVA conjugate was successfully synthesized by the reductive amination reaction. The conjugate was efficiently taken up by tumor cells, followed by the enhanced presentation of a model foreign antigen (OVA) as part of the MHC class I complex. Our findings based on a CMD-OVA polymeric conjugate as a foreign antigen-delivery system could serve as a platform technology for treatments of cancer.

## Abbreviations

CLSM: Confocal laser scanning microscopy
CMD: Carboxymethyl dextran
CTLs: Cytotoxic T lymphocytes
Cy5.5: Cyanine 5.5
EDC·HCl: 1-ethyl-3-(3-dimethylaminopropyl)carbodiimide·hydrochloride
FBS: Fetal bovine serum
FITC: Fluorescein isothiocyanate
MHC: Major histocompatibility complex
NaBH3CN: Sodium cyanoborohydride
NHS: N-hydroxysuccinimide
NIRF: Near-infrared fluorescent
OVA: Ovalbumin
